# Experimental Comparison of Radon Domain Approaches for Resident Space Object’s Parameter Estimation

**DOI:** 10.3390/s21041298

**Published:** 2021-02-11

**Authors:** Selenia Ghio, Marco Martorella, Daniele Staglianò, Dario Petri, Stefano Lischi, Riccardo Massini

**Affiliations:** 1 National Laboratory of Radar and Surveillance Systems (RaSS), Consorzio Nazionale Interuniversitario per le Telecomunicazioni (CNIT), 56124 Pisa, Italy; marco.martorella@iet.unipi.it; 2 Department of Information Engineering, University of Pisa, 56122 Pisa, Italy; 3 ECHOES srl, 56023 Cascina, Italy; daniele.stagliano@echoes-tech.it (D.S.); dario.petri@echoes-tech.it (D.P.); stefano.lischi@echoes-tech.it (S.L.); riccardo.massini@echoes-tech.it (R.M.)

**Keywords:** SSA, SST, Radon, autocorrelation, SIRTA, time-frequency, period estimation, rotating targets, radar, comparison, space debris, RSO, micro-Doppler

## Abstract

The fast and uncontrolled rise of the space objects population is threatening the safety of space assets. At the moment, space awareness solutions are among the most calling research topic. In fact, it is vital to persistently observe and characterize resident space objects. Instrumental highlights for their characterization are doubtlessly their size and rotational period. The Inverse Radon Transform (IRT) has been demonstrated to be an effective method for this task. The analysis presented in this paper has the aim to compare various approaches relying on IRT for the estimation of the object’s rotation period. Specifically, the comparison is made on the basis of simulated and experimental data.

## 1. Introduction

The increasing space activity for Earth observation in the last 50 years has led to the launch of many satellites and other space objects which orbit around the Earth [1,2,3,4,5,6,7,8]. Debris can adversely affect the functionality of satellites or other assets, including services such as earth observation, timing/navigation/positioning services, telecommunications, and space science. Resident Space Objects (RSOs) are represented by both active satellites and space debris in orbit around the Earth. Space debris are all man-made objects in Earth orbit or re-entering the atmosphere that are non-functional. The majority of these RSOs consists of debris, in particular inactive spacecrafts, parts of launch vehicles and fragments of systems created by explosions or collisions. Various assets also populate the space that perform a number of functions such as communication, earth observation, surveillance, navigation, etc. It is of vital importance to observe these assets, more specifically for their control in the case of friendly objects, and for gathering necessary intelligence in the case of unfriendly objects. Moreover, for the protection of current and future space missions, space debris must be continuously monitored and tracked in order to prevent catastrophic collisions. In fact objects in orbit around the Earth travel at a velocity of several km per second, which generate enough energy to completely destroy satellites [9]. Because of the high speeds of objects in orbit, even small pieces of debris can be very damaging in a collision. The impact of a 1-cm-sized debris on a spacecraft is approximately equivalent to the energy released by an exploding hand grenade. Low-Earth orbit is presently the most congested orbit. All majorly used orbits (LEO, MEO, HEO/GEO) will become more congested over time. Contributing factors include the rising commercial interest in space and the rapid increase in the use of small satellites such as picosats, nanosatellites/cubesats and microsats. The fragments created by a collision can drive a cascading process, known as the “Kessler syndrome” [10,11,12], in which each collision between objects generates more debris, therefore increasing the probability of further collisions. In order to protect valuable space assets, it is necessary to observe and monitor RSOs [13,14,15,16,17]. In this context, the availability of efficient methods and algorithms for accurate estimation of geometrical and motion parameters is extremely important. The use of Inverse Synthetic Aperture Radar (ISAR) images for space objects’ characterization is a valuable tool but a very large bandwidth is needed to achieve sufficient range resolution especially when dealing with centimeter objects [13,18,19]. For this reason, solutions based on time-frequency analysis [20] may be preferable when a large bandwidth is not available since they have the advantage of not requiring a large bandwidth.

Literature has reported on many studies concerning the period estimation by using radar [21,22,23,24,25,26,27].

A fast and easy way to estimate the period would be to use the autocorrelation function (AF). Nonetheless, as stated in [25], this method is not very robust to noise and leads to ambiguities when dealing with objects displaying symmetries. Both those issues are overcome by considering methods making use of the Inverse Radon transform (IRT). A prove of this can be found in [26,27], where the ability of the Inverse Radon transform (IRT) to estimate the rotation period in a robust way under different conditions is proven on the basis of simulations and experimental data, respectively. In fact, differently from the AF, the proposed methodology is able to solve ambiguities due to target’s symmetries, since it does not rely only on the frequency information, as in the case of AF but on the time-frequency joint information since it makes use of the spectrogram. In fact, when using a time-frequency transform, the algorithm is able to distinguish a cube rotating at ω from an object with 8 fold symmetry rotating at ω/2 since their response appear different.

In [21], the IRT has been also successfully used for the estimation of the time-frequency parameters with a gapped input signal. In [23,24], the IRT is applied to radar data from a helicopter for estimating the rotational speed of the blade. In particular, the results presented in [24], are preliminary results and therefore not accurate. The results reported in [23], although as stated by the authors themselves require adjustments, are valid and also include an analysis based on simulations.

The work presented in [25] still relies on the use of the IRT to estimate the period of rotation. Nevertheless, instead of exploiting the time-frequency signals, as in our approach [26,27] and in [21,22,23,24], it uses range-Doppler images whose resolution depends on the available bandwidth, unlike when using time-frequency methods. An additional difference with respect to the approach in [26,27], is represented by the use of the largest peak (LP) of the IRT instead of the concentration measure (CM).

By looking at the literature of methods for the period estimation that rely on IRT, we can notice that two main metrics are used for the period selection, i.e., [21,26,27] use the CM, whereas [23,24] use the largest peak (LP).

In order to compare different methods based on the use of IRT, this paper considers the combination of different methodologies proposed in [25,27] and compare their performance using simulated and experimental data. A preliminary comparison analysis based on simulation data only, has been presented in [28]. In particular, the following work extend the simulation analysis reported in [28] and validates the analysis using real data acquired in a controlled scenario. For the analysis, two different types of objects have been considered. Furthermore, during simulation, the two point scatterer models have been created according to the geometry of the real objects. Specifically, in this paper, the following methods will be evaluated:

1. SIRTA-CM: The Spectrogram Inverse Radon Transform based Algorithm—Concentration Measure (SIRTA-CM) is the algorithm introduced by the same authors in [27]. This algorithm to estimate the rotation period of the object exploits the IRT of the received signal. In particular, the period is estimated from the IRT displaying the maximum Concentration Measure (CM).
2. SIRTA-LP: SIRTA-LP is correspondent to SIRTA-CM except for the fact that instead of using the CM to estimate the period from the IRT, it considers the largest peak of the IRT.
3. AF-CM: Autocorrelation Function—Concentration Measure (AF-CM) relies on two main steps. In the first step the period is estimated by exploiting the autocorrelation function of the signal. However, since the AF-based estimation may suffer from ambiguities due to the level of noise or to the geometry of the object, a refinement step is added. This second step consist in applying the SIRTA-CM methodology on a smaller subset respect to full domain of candidate periods. The subset domain is constructed from the period estimated during the first step by taking values around it and around a number of its multiples.
4. AF-LP: AF-LP corresponds to AF-CM except for the fact that instead of using the CM to estimate the period from the IRT, it considers the largest peak of the IRT.

The rest of this paper is organized in the following manner. The model of the signal received by the radar is described in Section 2. In Section 3 the different approaches considered within this work are introduced. In Section 4 the results obtained by applying all the methods on both simulated and real data are shown and discussed. In Section 5, conclusions are drawn and further work is outlined.

## 2. Signal Model

In this section, we introduce the signal model by assuming a Frequency Modulated Continuous Wave (FMCW)-based radar. In particular, the model of the transmitted signal can be expressed as follows:

(1) $$s\left(t_{f}\right)=rect\left(\frac{t_{f}}{T_{r}}\right)\exp\left\{j2\pi \left[f_{0}t_{f}+\frac{1}{2}\mu t_{f}^{2}\right]\right\}$$

where $t_{f}$ is the fast-time, $T_{r}$ is the sweep repetition interval, $f_{0}$ is the central frequency and μ is the frequency sweep rate equal to the ratio of the transmitted bandwidth *B* and the sweep time. $T_{r}$. It must be specified that, the FMCW transmitted signal is a periodic signal with period equal to $T_{r}$. In particular, a generic time instant t can be expressed as:

(2) $$t=mT_{r}+t_{f}$$

where *m* represents the index of the $m^{th}$ transmitted sweep and $mT_{r}$ corresponds to the so-called slow-time $t_{m}$. Thus, the transmitted signal at the $m^{th}$ sweep becomes:

(3) $$\begin{array}{cc} s(t_{f},t_{m}) & =rect\left(\frac{t_{m}+t_{f}}{T_{ob}}\right)\\ & \exp\left\{j2\pi \left[f_{0}\left(t_{m}+t_{f}\right)+\frac{1}{2}\mu t_{f}^{2}\right]\right\} \end{array}$$

Let us consider an object rotating with a rotation rate ω and located at a range $R_{0}$. The received signal is a delayed version of the transmitted signal (the amplitudes are not considered in this derivation) and can be expressed as:

(4) $$\begin{array}{cc} s(t_{f},t_{m}) & =rect\left(\frac{t_{m}+t_{f}-t_{d}}{T_{ob}}\right)\\ & \exp\left\{j2\pi \left[f_{0}\left(t_{m}+t_{f}-t_{d}\right)+\frac{1}{2}\mu {\left(t_{f}-t_{d}\right)}^{2}\right]\right\} \end{array}$$

where

(5) $$t_{d}=\frac{2R_{P}\left(t_{f},t_{m}\right)}{c}$$

is the delay time, *c* is the speed of light and $R_{P}$ is the distance of a generic scatterer *P* of the target with respect to the radar.

The transmitted and received signals are mixed (dechirped) and low-pass filtered, in order to generate the beat signal:

(6) $$\begin{array}{c} s_{b}\left(t_{f},t_{m}\right)=rect\left(\frac{t_{m}+t_{f}-t_{d}}{T_{obs}}\right)\exp\{-j\frac{2\pi }{c}\left[2\mu R_{P}\left(t_{f},t_{m}\right)+2f_{c}R_{P}\left(t_{f},t_{m}\right)\right.\\ \left.-\frac{2\mu }{c}R_{P}{\left(t_{f},t_{m}\right)}^{2}\right]\} \end{array}$$

By taking the Fourier Transform (FT) of Equation (6) and neglecting the Residual Video Phase terms, we obtain the signal expressed in the beat frequency-slow time domain. Such a signal can be expressed as:

(7) $$\begin{array}{cc} S_{b}\left(f_{b},t_{m}\right) & =\sigma T_{obs}sinc\left[T_{obs}\left(f_{b}+\frac{2\mu }{c}R_{P}\left(t_{m}\right)\right)\right]\\ & \exp\left\{-j\frac{4\pi }{c}f_{c}R_{P}\left(t_{m}\right)\right\} \end{array}$$

**Figure 1:**
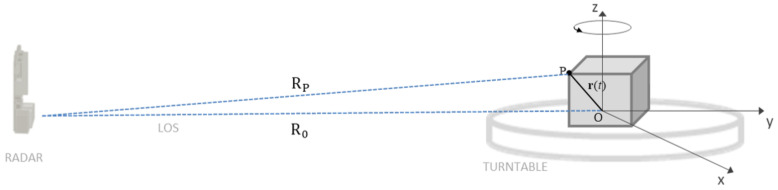
Observation geometry of an object rotating by means of a turntable.

Referring to the geometry in Figure 1, the overall motion projected onto the Line of Sight (LOS) can be expressed as:

(8) $$R_{P}\left(t_{m}\right)=R_{0}\left(t_{m}\right)+r\cos\left(\omega t_{m}+\phi_{0}\right)$$

where *r* is the distance of a generic scatterer *P* with respect to rotation center *O* embedded onto the target.

The total radial speed can be expressed as:

(9) $$v\left(t_{m}\right)=\frac{dR_{P}\left(t_{m}\right)}{dt}=-r\omega \sin\left(\omega t_{m}+\phi_{0}\right)$$

The Equation (7) is modulated in phase by $R_{P}\left(t_{m}\right)$, which leads to the generation of micro-Doppler effect in slow-time induced by the rotating scatterer. In the subsequent analysis, we will neglect the range information and “fuse” the signal along the range coordinate. By doing so, effects such as range cell migration can be completely ignored. The signal that we obtain is a slow-time signal $s_{R}\left(t_{m}\right)$ where the change of distance between the target’s scatterer and the reference point *O* generates a variation in the radial speed as shown in (9) and consequently a variation of the Doppler frequency, that can be expressed as:

(10) $$f_{D_{Rot}}\left(t_{m}\right)=\frac{2f_{0}}{c}\;\left[\omega \times r\right]\cdot {\hat{i}}_{LOS}$$

where, ${\hat{i}}_{\mathrm{LOS}}$ is the LOS unit vector, ω is the rotation velocity vector and r is the distance of *O* from the point *P*.

## 3. Periodicity Estimation Methods

In this section, the four different methods considered for the comparison are described.

### 3.1. SIRTA-CM

The Spectrogram Inverse Radon Transform based Algorithm—Concentration Measure (SIRTA-CM) is the algorithm introduced by the same authors in [27]. This algorithm to estimate the rotation period of the object exploits the IRT of the received signal. In particular, first the spectrogram of the signal received by the radar is computed. Then, the IRT of the spectrogram is calculated for all the candidate periods. Finally, the period is estimated from the IRT displaying the maximum Concentration Measure (CM). Before describing the algorithm in detail, some parameters need to be defined. Considering a generic rotating object, this will be characterized by a rotation period *T*. Referring to the observation time of the radar with $T_{ob}$, the number of periods contained in the observation time is defined as the ratio $T_{ob}/T$. In particular, we define the vector η=[η(1),η(2),...,η(Γ)] with elements spacing Δη as the vector spanning all candidates number of periods, where:

(11) $$\eta \left(\gamma \right)=\frac{T_{ob}}{T_{\gamma }}$$

where $T_{\gamma }$ is the $\gamma^{th}$ candidate rotation period with γ=[1,2,...,Γ] and Γ represents the number of candidate periods considered. In order to estimate the period, the IRT of the spectrogram is calculated over the elements of η. In particular the $\gamma^{th}$ IRT is calculates as follow:

(12) $${\mathrm{IR}}_{\gamma }\left(p,q\right)=\mathrm{IRT}\left(S_{i}\left(n,k\right),\alpha \left(\gamma \right)\right)$$

where, $\alpha \left(\gamma \right)=\theta_{set}\eta \left(\gamma \right)$ is a vector of angles with $\theta_{set}$ equally spaced vector of values between 0 and 2π. The length of $\theta_{set}$ is given by the spectrogram time-length. The definition of the spectrogram **S** can be found in Appendix B.

The Concentration Measure (CM) [29] reflects how the energy content is distributed in the Radon inverse domain. The maximum of the CM is obtained when the IRT is calculated using the number of rotations contained in the observation time η(γ). The CM for the γ-th candidate, η(γ), is given in (13).

(13) $$M_{\gamma }=\frac{{\sum_{p}\sum_{q}\left[{\mathrm{IR}}_{\gamma }\left(p,q\right)+\left|{\mathrm{IR}}_{\gamma }\left(p,q\right)\right|\right]}^{4}}{{\left\{{\sum_{p}\sum_{q}\left[IR_{\gamma }\left(p,q\right)+\left|IR_{\gamma }\left(p,q\right)\right|\right]}^{2}\right\}}^{2}}$$

**Figure 2:**
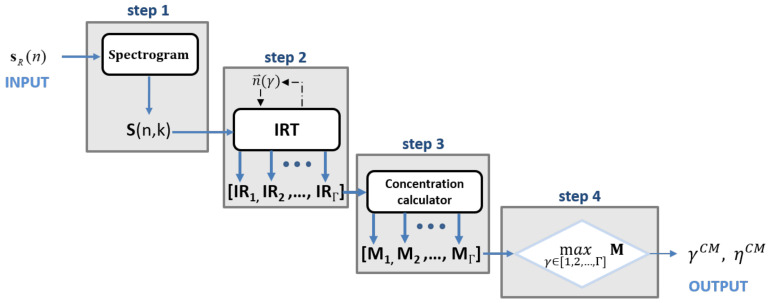
Spectrogram Inverse Radon Transform based Algorithm—Concentration Measure (SIRTA-CM) algorithm workflow.

In Figure 2, the SIRTA-CM’s flowchart is reported.

The definitions of the steps composing the algorithm shown in Figure 2 are the following:

- step 1: the spectrogram is calculated from the received signal $s_{R}\left(n\right)$ (in the discrete domain).
- step 2: the IRT is calculated for each value contained in the set of candidates values η
- step 3: the CM is calculated from (13) by considering each element of the set of values in **IR** = [${\mathrm{IR}}_{1},{\mathrm{IR}}_{2},...,{\mathrm{IR}}_{\gamma },...,{\mathrm{IR}}_{\Gamma }$].
- step 4: $\gamma^{CM}$ is found by searching for the index corresponding with the maximum value of the CM vector **M** = [$M_{1},M_{2},...,M_{\gamma },$ $...,M_{\Gamma }$].
  (14) $$\gamma^{CM}=\arg\max_{\gamma \in [1,2,...,\Gamma ]}M_{\gamma }$$

At last, the SIRTA-CM algorithm returns the estimated number of observed rotation periods $\eta^{CM}$:

(15) $$\eta^{CM}=\eta \left(\gamma^{CM}\right)$$

from this value, the IRT associated with the estimated number of observed periods can be calculated as follows:

(16) $$\mathrm{IR}\left(p,q\right)=\mathrm{IRT}\left(S\left(n,k\right),\alpha^{CM}\right)=\mathrm{IRT}\left(S\left(n,k\right),\theta_{set}\eta^{CM}\right)$$

and the rotation period can be expressed as:

(17) $${\tilde{T}}_{SIRTA-CM}=\frac{T_{ob}}{\eta^{CM}}$$

### 3.2. SIRTA-LP

The methodology SIRTA-LP corresponds to SIRTA-CM except for the fact tha instead of using the CM to estimate the period from the IRT, it considers the largest peak (LP) of the IRT.

**Figure 3:**
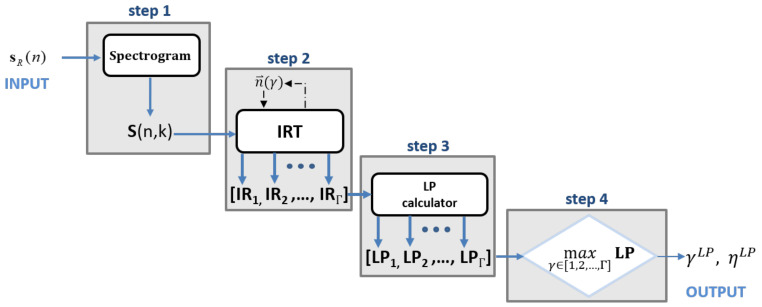
SIRTA-LP algorithm workflow.

The SIRTA-LP workflow is shown in Figure 3.

The SIRTA-LP can be described as follows:

- step 1: the spectrogram is calculated from the received signal
- step 2: the IRT is calculated for each value contained in the set of candidates values η
- step 3: the LP for all the IRT images contained in the set **IR** = [${\mathrm{IR}}_{1},{\mathrm{IR}}_{2},...,{\mathrm{IR}}_{\gamma },...,{\mathrm{IR}}_{\Gamma }$] is derived and stored in the vector **LP** = [${\mathrm{LP}}_{1},{\mathrm{LP}}_{2},...,{\mathrm{LP}}_{\gamma },$ $...,{\mathrm{LP}}_{\Gamma }$].
- step 4: $\gamma^{LP}$ is found by searching for the index corresponding to the maximum of the vector **LP**.
  (18) $$\gamma^{LP}=\arg\max_{\gamma \in [1,2,...,\Gamma ]}{\mathrm{LP}}_{\gamma }$$

At last, the SIRTA-LP algorithm return the estimated number of observed rotation periods $\eta^{LP}$:

(19) $$\eta^{LP}=\eta \left(\gamma^{LP}\right)$$

from this value, the IRT associated with the estimated number of observed periods can be calculated as follow:

(20) $$\mathrm{IR}\left(p,q\right)=\mathrm{IRT}\left(S\left(n,k\right),\alpha^{LP}\right)=\mathrm{IRT}\left(S\left(n,k\right),\theta_{set}\eta^{LP}\right)$$

and the rotation period can be expressed as:

(21) $${\tilde{T}}_{SIRTA-LP}=\frac{T_{ob}}{\eta^{LP}}$$

### 3.3. AF-CM

The Autocorrelation Function—Concentration Measure (AF-CM) algorithm exploits the autocorrelation function of the received signal to estimate the rotation period of the RSO. In particular, the AF-CM method relies on two main steps. In the first step the period $T_{AF}$ is estimated by exploiting the autocorrelation function of the signal. However, since the AF-based estimation may suffer from ambiguities due to the level of noise or to the geometry of the object, a refinement step is added. This second step consists in applying the SIRTA-CM methodology on a smaller subset respect to full domain of candidate periods. The subset domain is constructed from the period estimated during the first step $T_{AF}$ by taking values around it and around a number of its multiples. In particular, the CM of the IRT is evaluated for a vector defined around $\eta^{AF}=T_{ob}/T_{AF}$ and its integer multiples. The search range can be set to $\eta \in [\left[a\left(1\right)*\eta^{AF}-\Delta N,...,\Delta \eta ,...,a\left(1\right)*\eta^{AF}+\Delta N\right],...,\left[a\left(n\right)*\eta^{AF}-\Delta N,...,\Delta \eta ,...,a\left(n\right)*\eta^{AF}+\Delta N\right]]$, where ΔN is a small value respect to $\eta^{AF}$ and a is a vector of integer values. At the end of the procedure $T_{AF-CM}$ and $\eta^{AF-CM}$ are returned. In this work the values a=[1,2,...6] are considered.

What makes this method different from SIRTA-CM is mainly represented by the fact that introducing this initial step reduces the search domain on which to calculate the IRT. Since the calculation of IRT is the most expensive operation of the SIRTA algorithm, reducing the number of times the IRT is calculated reduces the overall cost of the algorithm.

### 3.4. AF-LP

The AF-LP method corresponds to AF-CM except for the fact that instead of using the CM to estimate the period from the IRT, it considers the largest peak of the IRT.

## 4. Performance Comparison Analysis

In the following sections, the comparison analysis by using both simulated and real data is performed in terms of the Normalized Root Mean Square Error (NRMSE). The definition of the NRMSE can be found in Appendix A.

### 4.1. Simulation Set Up

The parameters used for the simulation are shown in Table 1.

In particular, complex additive white Gaussian noise (AWGN) is considered to take into account the effect of the Signal to Noise Ratio (SNR) on the performances of the four methods when the data are affected by high levels of noise. For instance, the the following SNR levels are used: {−5, 0, 5, 10, 15, 20} dB and to better generalize for each value 100 realizations of noise are generated.

For the sake of simplicity, the point scatterer model, widely used in radar applications, is assumed to model the target. More in detail, the point scatterer model, assumes that the electromagnetic backscattered signal from a complex object can be approximated by the backscattering from a set of scattering centers on the object.

To simplify the simulation, the only the rotation motion is taken into account and no translational motion is included.

**Figure 4:**
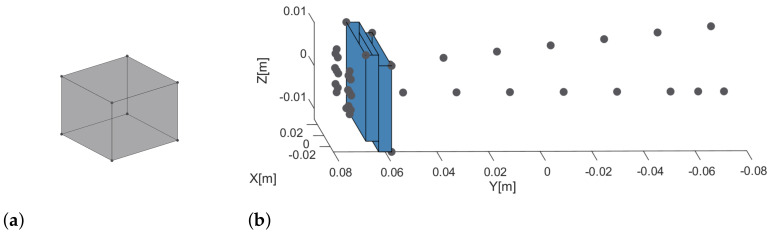
Simulated objects. (**a**) point scatterer model of the first object; (**b**) point scatterer model of the second object.

The first simulated object is the point scatterer model of a cube with 10.2 cm side, whose size is in agreement with the typical size of a CubeSat, see Figure 4a. The second object Figure 4b. considered during simulation, is the point scatterer model of the second actual object shown in Figure 6 that is an object created by the composition of two bolts, two screws and two metal plates.

**Figure 5:**
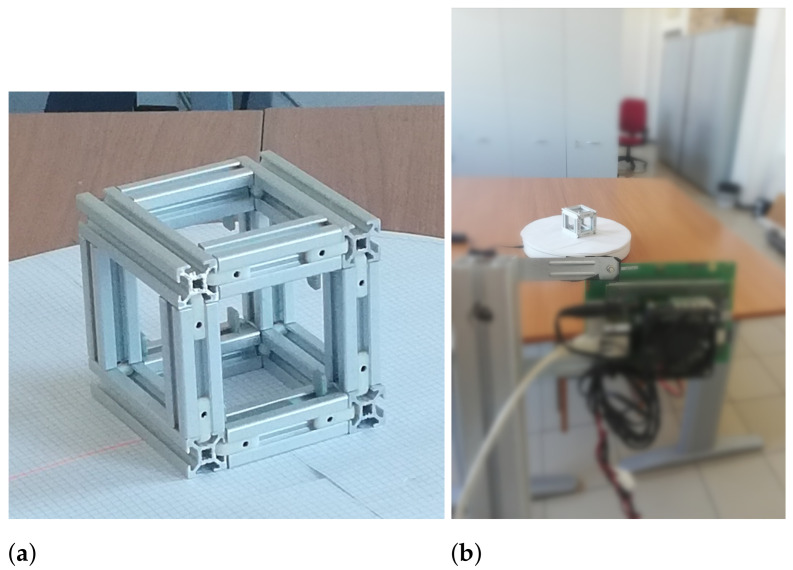
First object. (**a**) Photo of the object used as target during experiments; (**b**) photo of the acquisition set-up of the experiment.

**Figure 6:**
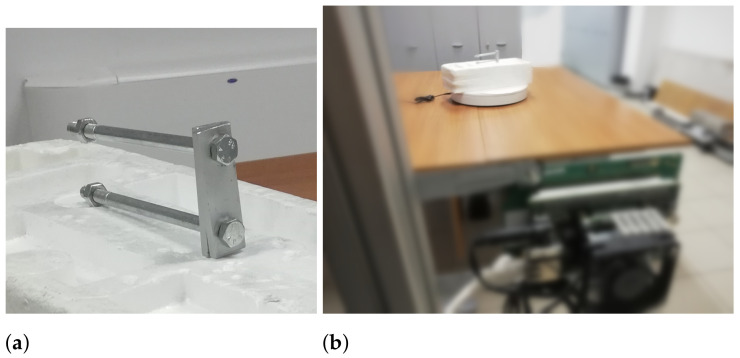
Second object. (**a**) Photo of the object used as target during experiments; (**b**) photo of the acquisition set-up of the experiment.

In particular, during simulation, the point scatterer model of the real objects used during measurements has been created. The figures of the real objects are reported later in the paper, see Figure 5 and Figure 6.

During simulations, the objects rotates about the axis [0,0,1] with a period of 7.2 s and ${\hat{i}}_{LOS}=\left[0,\;1,\;0\right]$. The SIRTA method has been successfully tested also considering different rotating axes from the one orthogonal to the LOS, more details can be found in [30].

### 4.2. Simulation Results

In the following section, the results will be displayed and discussed for comparison. The NRMSE values obtained by using the four methodologies fot the two simulated RSOs are shown in Table 2 and Table 3, respectively. In particular, the following results were obtained using Δη=0.01 and a=6. As expected, the use of the AF to estimate the periods led to ambiguous results, also with high SNR, since the first simulated object was symmetric. This happened also considering the second object since, even if it was not perfectly symmetric, it was rotated around its center of symmetry.

Differently, by introducing the refinement step as in AF-CM/LP the ambiguity was resolved for both the simulated cases.

Specifically, all the methodologies considered for the comparison displayed good performances with both objects. In particular, the best result was obtained using SIRTA-CM in both cases.

From both Table 2 and Table 3, we can notice that increasing the SNR from 15 to 20 dB did not influence the result considerably, this may be due to a saturation effect. Let us clarify that in Table 2 and Table 3 the NRMSE happened to be 0 because the exact rotation speed of the object coincided with one of the values in the list of candidate periods.

In terms of computational time on a general purpose computer (Intel Core i7-6700 CPU @ 3.4 GH and a 32 GB RAM), using Δη=0.01 and a=6, SIRTA-CM methodology required about 140 s to be executed. Differently, AF-CM methodology required only about 8 s to be executed. This is due to the fact that SIRTA-CM worked on the full domain, and instead AF-CM operated on a sub-domain. For this reason, since for both the tested objects the AF-CM method displayed a good accuracy, if a faster approach is needed, AF-CM may be considered an alternative option. However, this choice may affect the goodness of the estimation. In fact, when the initial guess of the period is close to a multiple or a fraction of the actual one, the AF-based algorithms had poor performances.

### 4.3. Experimental Set-Up

Real experiments were carried out in collaboration with Echoes s.r.l. In particular, the radar used was a K-band FMCW radar with a direct deramping architecture. During the experiments, the radar was transmitting a FMCW signal at the frequency of 23.5 GHz with a bandwidth of 1 GHz and a transmitted power of 6 dBm. Additionally, we used a programmable rotating platform capable of azimuthal rotation. The turntable system was used to emulate the motion of a space debris. The distance from the imaging radar to the turntable’s center was 1.5 m, and the radar LOS was perpendicular to the object’s spin vector. Two objects have been measured. The first object is a cube with 10.2 cm side, which size is in agreement with the typical size of a CubeSat. In Figure 5 we can see a photo of the actual object used during experiments and its point like scatterers model.

The second object is a composition of two bolts, two screws and two metal plates, see Figure 6. The object’s maximum length is 14.5 cm.

### 4.4. Experimental Results

The results obtained by applying the four considered methodologies to the acquired data are shown and discussed here. The NRMSE values of the estimated rotation period obtained using the four methodologies are shown in Table 4. In particular, Table 4 shows the results obtained using Δη=0.01. As already noticed for the simulation results, also when dealing with real data the use of the AF led to ambiguities since both objects rotated around their point of symmetry. From the first line of Table 4, relative to the cube, we can observe a good agreement with the result obtained from the simulations. Thus, the comments drawn from the simulation results also apply to the experimental results. The second line of Table 4, reports the results obtained measuring the second object. In this case, we can observed that in pairs, the four methods, had the same performances. Thus, for this particular type of object, CM and LP could be both used effectively.

## 5. Conclusions

In this work, four methodologies that are based on the use of IRT, for the estimation of the rotation period of RSOs, have been compared. The comparison has been made on the basis of simulated and experimental data. By analysing the results, we have seen that among all the methods considered the SIRTA-CM is the one showing the best performances in term of robustness and accuracy. Even so, considering that SIRTA-CM works on the full domain instead AF-CM on a sub-domain, from the analysis shown in this paper the AF-CM may be considered an alternative option if a faster approach is needed. However, this choice may affect the goodness of the estimation. In fact, when the initial guess of the period is close to a multiple or a fraction of the actual one, the AF-based algorithms have poor performances. It is in the interest of the authors to test these algorithms on radar data of actual space debris if they will become available in the future. 

## Figures and Tables

**Table 1:** Simulation parameters.

Symbol	Description	Value
$f_{0}$	Carrier frequency	23.5 GHz
$T_{ob}$	Observation Time	7.2 s
$f_{s}$	Sampling frequency	100 Hz
$T_{i}$	Pulse Width	0.125 μs
$T_{r}$	Pulse Repetition Interval	10 ms

**Table 2:** Normalized Root Mean Square Error (NRMSE) for the first object.

SNR [dB]	SIRTA-CM	SIRTA-LP	AF-CM	AF-LP	AF
−5	0.0202	0.4051	0.0194	0.2208	2.0116
0	0.0127	0.0285	0.0139	0.0292	2.0115
5	0.0053	0.0311	0.0092	0.0304	2.0115
10	0	0.0352	0.0044	0.0336	2.0115
15	0	0.0410	0.0037	0.0394	2.0115
20	0	0.0471	0.0037	0.0449	2.0115

**Table 3:** NRMSE for the second object.

SNR [dB]	SIRTA-CM	SIRTA-LP	AF-CM	AF-LP	AF
−5	0.1371	0.7943	0.8351	2.3723	1.7843
0	0.0106	0.0359	0.0117	0.6240	1.3392
5	0.0026	0.0217	0.0091	0.0195	1.3404
10	0	0.0245	0.0069	0.0201	1.3408
15	0	0.0258	0.0063	0.0212	1.3412
20	0	0.0271	0.0041	0.0254	1.3405

**Table 4:** NRMSE with Δη=0.01.

Object	SIRTA-CM	SIRTA-LP	AF-CM	AF-LP	AF
First object	0	0.0620	0.0039	0.0582	1.6102
Second object	0	0	0.0548	0.0548	1.4675

## Abbreviations

The following abbreviations are used in this manuscript:

CM	Concentration Measure
FMCW	Frequency Modulated Continuous Wave
IRT	Inverse Radon Transform
ISAR	Inverse Synthetic Aperture Radar
LOS	Line Of Sight
LP	largest peak
NRMSE	Normalized Root Mean Square Error
PRI	Pulse Repetition Interval
RMSE	Root Mean Square Error
SIRTA	Spectrogram - Inverse Radon Transform based Algorithm
SSA	Space Situational Awareness
STFT	Short Time Fourier Transform

## Appendix A

### Appendix Normalized Root Mean Square Error

The *Root Mean Square Error* (RMSE) is commonly used to to analyse the error of an estimator. The RMSE can be defined as follows:

(A1) $$RMSE=\sqrt{\frac{1}{N_{r}}\sum_{i=1}^{N_{r}}{\left(x-{\hat{x}}_{i}\right)}^{2}}$$

where $N_{r}$ represents the number of noise realizations, *x* is the actual value and $\hat{x}$ is the estimated value.

By normalizing the RMSE, the comparison between datasets or models can be done independently of the scale factor.

(A2) $$NRMSE=\frac{RMSE}{x}$$

## Appendix B

### Appendix Spectrogram Definition

The spectrogram (**S**(t,f)) of $s_{R}\left(t\right)$ is defined as the squared modulus of its Short-Time Fourier Transform (STFT).

(A3) $$S\left(t,f\right)={\left|\mathrm{STFT}(t,f)\right|}^{2}$$

where

(A4) $$\mathrm{STFT}\left(t,f\right)=\int s_{R}\left(\tau \right)\;h\left(t-\tau \right)\;e^{-j2\pi f\tau }d\tau$$

and h(t) is the analysis window. In the discrete domain it becomes

(A5) $$S\left(n,k\right)={\left|\mathrm{STFT}(n,k)\right|}^{2}$$

where

(A6) $$\mathrm{STFT}\left(n,k\right)=\sum_{m=0}^{N_{h}-1}h\left(m\right)\;s_{R}\left(n+m\right)\;e^{-j\frac{2\pi }{N_{h}}mk}$$

and h(n) is the discrete analysis window of length $N_{h}$.
